# Regional anaesthesia-based free flap reconstruction for limb salvage in high-risk patients with refractory lower limb infections

**DOI:** 10.1016/j.jpra.2025.01.005

**Published:** 2025-01-09

**Authors:** Mitsutoshi Ota, Makoto Motomiya, Marie Okada, Ryo Miyashita, Naoya Watanabe, Norimasa Iwasaki

**Affiliations:** a Department of Orthopaedic Surgery, Obihiro Kosei hospital Hand Center, Obihiro, Japan; b Department of Orthopaedic Surgery, Faculty of Medicine and Graduate School of Medicine, Hokkaido University, Sapporo, Japan; c Department of Anaesthesiology, Obihiro Kosei Hospital, Obihiro, Japan

**Keywords:** Combined spinal-epidural anaesthesia, Free flap, High-risk patients, Lower limb salvage, Infection

## Abstract

**Background:**

Patients with severe comorbidities and refractory lower leg and foot infections face high risks from prolonged anaesthesia and complex soft tissue reconstruction. Our institution collaborates with anaesthetists to perform limb salvage using free flaps, primarily under combined spinal-epidural anaesthesia (CSE) without general anaesthesia (GA). This study aimed to evaluate the treatment outcomes of our limb salvage algorithm in high-risk patients.

**Materials and methods:**

Between January 2020 and December 2023, we included patients with ASA class III or higher undergoing limb salvage for chronic osteomyelitis or diabetic gangrene, who desired limb preservation, had palpable main arteries and no urgent cardiovascular conditions. We investigated 12 patients with 13 limbs and 14 free flaps who underwent infection control and free flap reconstruction under CSE without GA.

**Results:**

Among the 14 free flaps, 9 were ASA class III and 5 were class IV. The median anaesthesia time was 562 min and median surgical time was 479 min. All flap surgeries, except for one, required no vasopressor usage to control intraoperative hypotension. Partial necrosis occurred in 2 flaps, but all flaps survived. One limb with recurrent osteomyelitis required a vascularised fibula graft. No severe systemic complications were observed, and all limbs were preserved with weight-bearing function in 11 of 13 limbs (85%).

**Conclusions:**

Our treatment algorithm using CSE without GA for severe lower limb infections demonstrates that limb salvage can be safely achieved by preventing flap necrosis and systemic complications.

## Introduction

Major amputations are often performed for chronic osteomyelitis and diabetic gangrene in the lower legs and feet; however, it can adversely impact the functional outcomes and survival prognosis, thus making limb preservation a critical goal.^1^, ^2^, ^3^, ^4^ Salvaging infected limbs typically involves extensive debridement, antibiotic therapy and free flap reconstruction.^1^, ^2^, ^3^ High-risk patients with poor vascular conditions are particularly susceptible to flap failure, although stable outcomes have been achieved using end-to-side (ETS) anastomosis for arteriosclerotic vessels.^3^^,^^5^, ^6^, ^7^ Prolonged general anaesthesia (GA) in high-risk patients can cause systemic complications, including fatal outcomes.^8^, ^9^, ^10^, ^11^ Consequently, treatment plans must address local reconstruction and systemic postoperative risks.^12^^,^^13^

Surgeries performed under regional anaesthesia alone are being increasingly used in high-risk patients to prevent GA-related systemic complications.^8^^,^^14^ Although lower limb free flap procedures have traditionally relied on GA, recent studies have demonstrated the efficacy of long-duration surgeries using only regional anaesthesia for free flap reconstruction.^15^^,^^16^ Despite the proven benefits of regional anaesthesia-based free flaps in high-risk patients, few studies have integrated this approach into limb salvage treatments for infected lower limbs. Limb salvage for refractory infections requires a multidisciplinary team, including plastic surgeons, vascular surgeons, orthopaedic surgeons, cardiologists, diabetologists and infectious disease specialists.^1^^,^^3^ However, no existing reports detail an anaesthesia-based algorithm for such cases. To address this gap, we developed a multidisciplinary algorithm in collaboration with anaesthetists to preserve weight-bearing limbs in high-risk patients.

The purpose of this study was to investigate the outcomes in high-risk patients with refractory lower limb and foot infections treated using our regional anaesthesia-based limb salvage algorithm without GA.

## Patients and methods

This study was approved by our institutional ethics committee (Approval No. 2023–104). It included patients with refractory osteomyelitis or gangrene of the legs or feet who were at high anaesthetic risk and treated according to our limb salvage protocol from January 2020 to December 2023. A retrospective analysis was conducted on cases classified as ASA class III or higher, based on the latest ASA-PS Classification System.^17^

**Figure 1:**
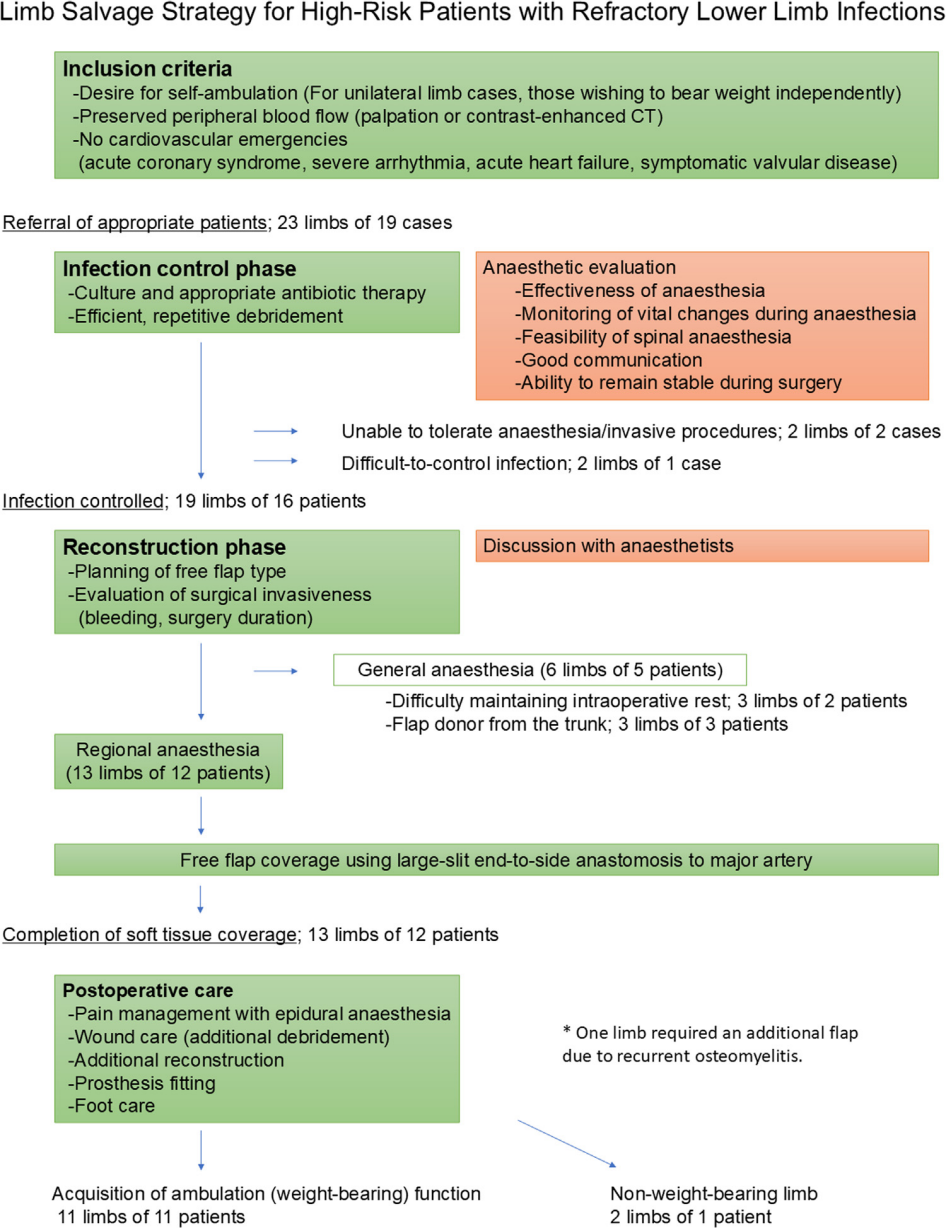
Algorithm for limb salvage strategy for high-risk patients with refractory lower limb infections.

Our institution's basic limb salvage algorithm for high-risk anaesthesia cases is shown in Figure 1. The inclusion criteria were the desire for self-ambulation, preserved main arterial circulation of the foot, and absence of acute cardiovascular diseases.

### Infection control phase

**Figure 2:**
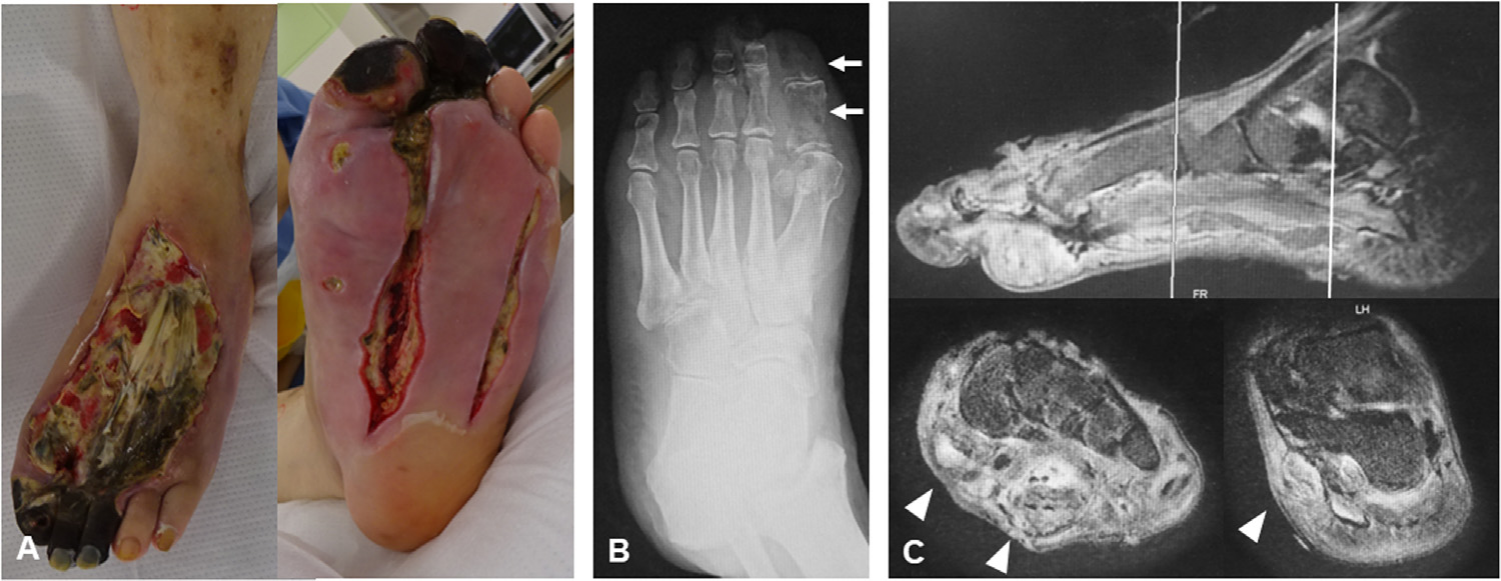
Infection control phase (case of a 78-year-old female with diabetic foot gangrene). (A) Photograph showing the initial external appearance at the first visit. Despite multiple debridements at another hospital, infection was not controlled, leading to a referral to our department for consideration of major amputation. (B) Radiograph highlighting osteolysis of the great toe bones (arrow). (C) MRI T2 fat-suppressed image demonstrating extensive infection along the plantar aspect of the foot, extending centrally along the flexor tendons (arrowhead).

**Figure 3:**
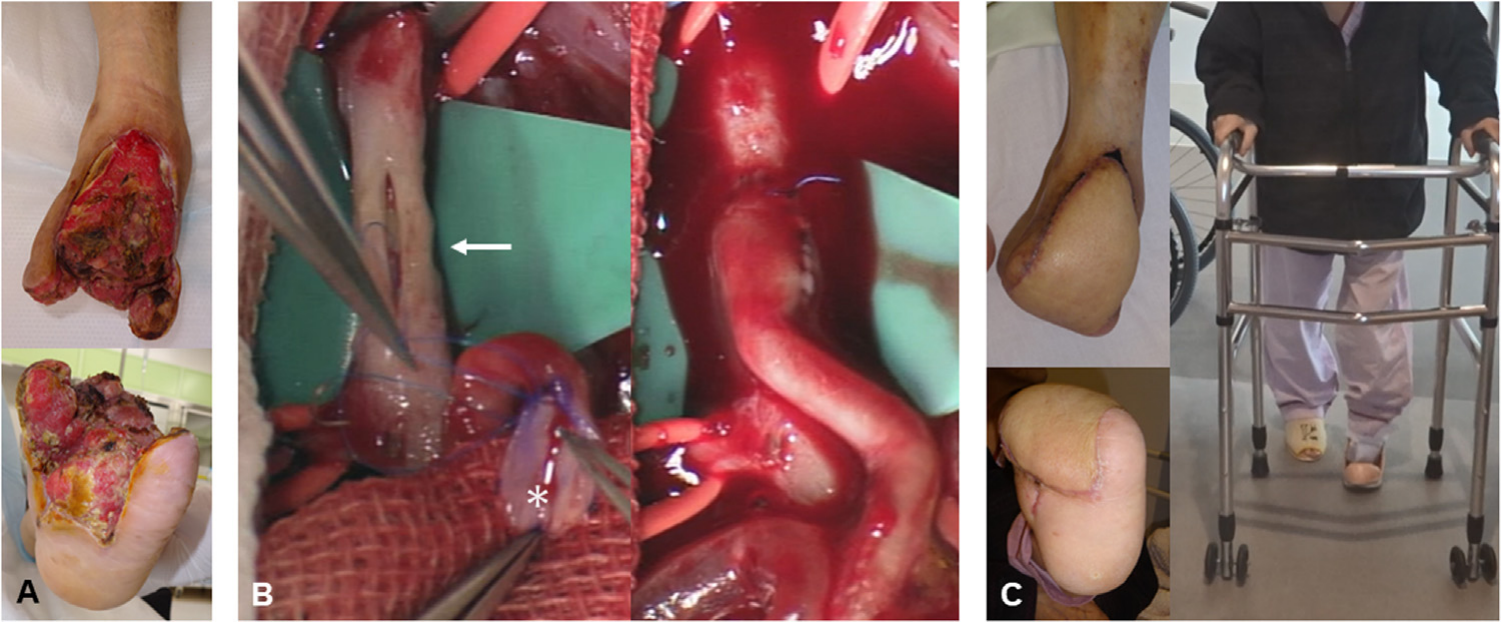
Reconstruction phase (same case as Figure 2). (A) Pre-reconstruction appearance after achieving infection control through multiple debridements. (B) Reconstruction using an anterolateral thigh flap under combined spinal-epidural anaesthesia. The flap's vessels (asterisk) are prominently enlarged, and arterial anastomosis was performed using the microscopic parachute end-to-side technique with large-slit vesselotomy (arrow) to the recipient vessel. (C) No systemic or flap-related complications were observed. Custom orthotics were fabricated approximately 1 month after surgery, and walking exercises were initiated during postoperative care.

The extent of infection was assessed through clinical evaluation and imaging, followed by extensive debridement (Figure 2). For patients on multiple anticoagulants, adjustments were made to manage bleeding risks prior to radical debridement. Antibiotic treatment was initiated with broad-spectrum antibiotics and adjusted based on pathogen sensitivity, in consultation with an infectious disease specialist. The efficacy of anaesthesia and any vital sign changes during debridement were closely monitored by considering the potential for subsequent free flap reconstruction. Once the local symptoms improved and tissue cultures returned negative results, treatment was advanced to the reconstruction phase (Figure 3A).

### Reconstruction phase

The reconstruction plan, including the method, expected surgery duration, estimated blood loss and flap harvest site, was developed in consultation with anaesthetists. Donor sites from the lower limb were primarily used under combined spinal-epidural anaesthesia (CSE) to minimise systemic complications. When cases requiring trunk flap harvests or regional anaesthesia posed challenges, reconstructions were performed under GA, and these cases were excluded from further analysis. Patients were informed of the elevated risk of systemic and flap-related complications compared to standard surgeries, and informed consent was obtained.

### Anaesthesia methods

Anaesthesia was administered using the needle-through-needle technique at L2/3 or L3/4 vertebral levels.^18^ For spinal anaesthesia, 10 to 20 mg of dissolved tetracaine was delivered, with fentanyl or morphine added depending on the patient's medical history. Following the injection, a catheter was inserted into the epidural space. The anaesthetist frequently monitored the anaesthetic level, and 0.2% to 0.5% ropivacaine was administered as required through the epidural catheter, with doses tailored to the patient's condition. Sedation with dexmedetomidine was administered when necessary.

### Surgical techniques

To ensure adequate blood supply to the flap, a major artery with good flow and adjacent deep veins were selected as recipient vessels. Whenever feasible, all branches at the anastomosis site were preserved to minimise disruption to peripheral and surrounding circulation. Vascular anastomoses were performed using the microscopic parachute end-to-side (MPETS) technique with a large-slit ETS anastomosis (Figure 3B).^19^^,^^20^

### Postoperative care

The epidural catheter was maintained for 3–5 days postoperatively, with a continuous infusion of 0.2% ropivacaine at a rate of 2–4 ml/hr. Bed rest was recommended during the first postoperative week, and smoking was strictly prohibited until the wound had fully epithelialised. Active muscle strengthening exercises commenced approximately one week after surgery, once the flap blood flow was stabilised. Orthotic devices were designed and fitted after the wound epithelialised, and weight-bearing walking training was initiated thereafter (Figure 3C).

### Evaluation items

We assessed patient demographics, flap details and treatment outcomes from cases in which infection control was achieved and free flap reconstruction was performed. Preoperative aesthetic risk evaluations included the Charlson comorbidity index (CCI) for systemic comorbidities,^21^ Revised cardiac risk index (RCRI) for cardiovascular risk in non-cardiac surgery patients,^22^ and Assess Respiratory Risk in Surgical Patients in Catalonia Preoperative Pulmonary Score (ARISCAT PPS) for respiratory complication risk.^23^ The extent of recipient vessel calcification was classified using the criteria established by Ferraresi et al.^24^ Postoperative complications were categorised using the Clavien-Dindo classification.^25^ Final lower limb function was assessed using the lower extremity functional scale (LEFS).^2^ Owing to the small sample size and non-normal distribution, all data were presented as median (IQR).

## Results

During the study period, 19 patients with 23 limbs met the inclusion criteria and were deemed eligible for reconstruction. In the infection control phase, 4 limbs from 3 patients underwent major amputation (Figure 1).

### Patient demographics and flap details

Infection control was successfully achieved in 16 patients with 19 limbs, all of whom progressed to the reconstruction phase. However, 5 patients with 6 limbs were excluded due to GA use. In one limb requiring reconstruction with a latissimus dorsi flap for calcaneal osteomyelitis, the anaesthetist recommended switching to a donor site that was manageable under CSE without GA due to high anaesthetic risks. Consequently, the reconstruction was performed using the vastus lateralis myocutaneous flap from the same side. The final cohort comprised 12 patients with 13 limbs, including 10 men and 2 women, with a median age of 60 years (Table 1). The aetiologies included foot gangrene in 6 limbs and osteomyelitis in 5 limbs. The most commonly used flap type was the anterolateral thigh (ALT) flap, which was employed in 11 cases. Anaesthesia-related complications are summarised in Table 2.

**Table 1:** Patient demographics and flap details.

Number of patients		12 patients
Number of limbs		13 limbs
Number of flaps		14 flaps

Sex	Men	10 patients
	Women	2 patients
Age, years		60 (52–74)
Body mass index (kg/m²)		23 (21–26)

Aetiology	Osteomyelitis	
	- Tibia	2 limbs
	- Calcaneus	2 limbs
	- Tarsal bone	1 limb
	Foot gangrene	6 limbs
	Necrotising fasciitis	1 limb
	Pyogenic foot arthritis	1 limb

Reconstruction site	Middle third of lower leg	1 limb
	Distal third of lower leg	3 limbs
	Hind foot	3 limbs
	Middle foot	6 limbs

Number of pre-flap debridement		5 (3–7) operations
	Partial amputation of foot	5 limbs

Flap type	Anterolateral thigh flap	11 flaps
	Vastus lateralis myocutaneous flap	2 flaps
	Vascularised fibula bone flap	1 flap

Donor flap side	Ipsilateral	9 flaps
	Contralateral	5 flaps

Data are presented as median (IQR).

**Table 2:** Medical conditions affecting anaesthesia.

Number of patients		12 patients

Comorbidities		
	ASO (revascularisation)	5 (1 EVT, 1 bypass)
	DM (haemoglobin A1c, %)	10 (9.0 (6.7–9.4))
	Uncontrolled DM (haemoglobin A1c > 8)	7
	HT	8
	HL	3
	CKD (creatinine; mg/dL)	8 (4.1 (1.6–7.1))
	HD	4
	Hypoalbuminaemia (albumin; g/dL)	11 (2.8 (2.1–3.0))
	CHD (history of PCI)	4 (1)
	COPD	4
	Anaemia (haemoglobin; g/dL)	11 (8.9 (8.2–10.2))
	Cerebral infarction	2

Smoking		4
Oral anticoagulant		3

Data are presented as median (IQR).

**ASO**, arteriosclerosis obliterans; **EVT**, endovascular treatment; **DM**, diabetes mellitus; **HT**, hypertension; **HL**, hyperlipidaemia; **CKD**, chronic kidney disorder; **HD**, haemodialysis; **CHD**, chronic heart disorder; **COPD**, chronic obstructive pulmonary disease.

### Reconstruction phase details

Details of anaesthesia and perioperative variables for free flap surgeries are presented in Table 3. Nine flaps in 8 patients were classified as ASA class III, and 5 flaps in 4 patients were classified as ASA class IV. Debridement was performed under spinal anaesthesia in nearly all cases. The median anaesthesia time was 562 min, median surgery time was 479 min and median ischaemia time was 132 min. All patients remained comfortable and free from pain or anxiety throughout the operative period under CSE.

**Table 3:** Anaesthesia details and perioperative variables in free flap surgery.

Number of flaps		14 flaps in 12 patients
ASA class	Class III	9 flaps in 8 patients
	Class IV	5 flaps in 4 patients

Charlson comorbidity index		4.0 (2.0–5.3)
	0	0
	1–2	5 patients
	3–4	2 patients
	≥5	5 patients

Revised cardiac risk index		1.5 (1.0–3.0)
	0	2 patients
	1	4 patients
	2	2 patients
	≥3	4 patients

ARISCAT PPS		37 (32–45)
	Low	1 patient
	Intermediate	7 patients
	High	4 patients

Anaesthesia for debridement*	Block	8 flaps
	Spinal	13 flaps
	GA	3 flaps

Anaesthesia time (minutes)		562 (534–631)
Operation time (minutes)		479 (453–536)
Flap ischaemia time (minutes)		132 (116–177)
Blood loss (ml)		134 (91–192)

Flaps with additional peripheral nerve blocks		1 flap
Flaps with dexmedetomidine for sedation management		12 flaps
Flaps with vasopressors to control intraoperative hypotension		1 flap
(Only 0.1 mg phenylephrine)		
Flaps with intraoperative transfusions		3 flaps

Data are presented as median (IQR). **ASA class**, American Society of Anesthesiologists classification; **ARISCAT PPS**, assess respiratory risk in surgical patients in Catalonia preoperative pulmonary risk score; **CSE**, combined spinal-epidural anaesthesia; **GA**, general anaesthesia*: There are duplicate records in the table.

**Figure 4:**
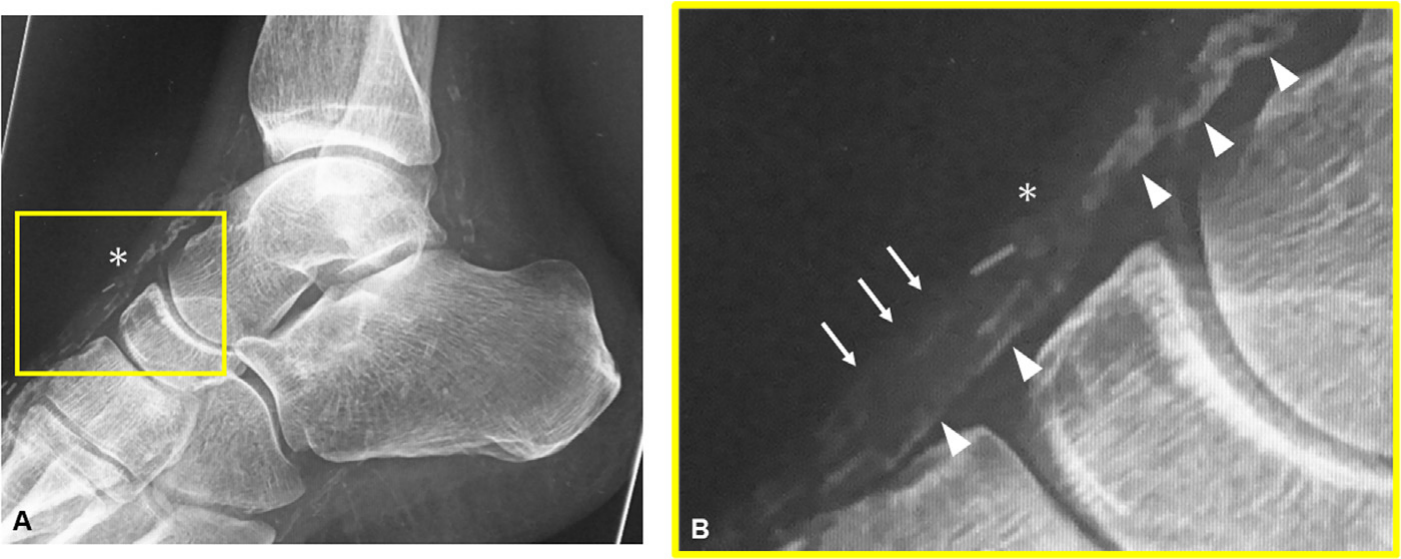
(A, B) (zoomed photos): Postoperative X-ray of a case showing calcification in the flap and recipient vessels. The interruption of calcification in the dorsalis pedis artery (arrowhead) indicates the anastomotic site (asterisk), where the calcified pedicle vessel of the anterolateral thigh flap (arrow) was anastomosed using the microscopic parachute end-to-side technique.

No arterial spasm was observed at the recipient artery during anastomosis. Stable vital control was maintained, preventing critical intraoperative hypotension, without using vasopressors in all but one case. Details of the vascular anastomosis are provided in Table 4. In cases where the recipient and flap vessels exhibited calcification, the recipient vessel was selected to minimise calcification, and MPETS anastomosis was performed (Figure 4).

**Table 4:** Anastomosis details.

Number of flaps		14 flaps

Recipient artery	Tibialis posterior	6
	Tibialis anterior	2
	Dorsalis pedis	6

Flaps with recipient arterial calcifications		10
Medial arterial calcification score*		1 (0–4)
Flaps with pedicle calcifications		6

Number of venous anastomoses	1	5
	2	9

Recipient vein	Accompanying vein	14

Data are presented as median (IQR).

⁎ According to the Frerraresi's classification.^26^

### Flap outcomes and complications

Surgery-related complications were observed in 7 cases; however, all flaps survived (Table 5). Systemic complications related to anaesthesia were monitored up to 30 days postoperatively. One flap developed asymptomatic deep vein thrombosis on postoperative day 17, which required oral anticoagulant therapy. No cases of delirium or severe systemic complications were observed. Flap-related complications included partial necrosis in 2 flaps. One case involved distal necrosis of a large ALT flap beyond the angiosome, which was managed with additional debridement and epithelialisation. The second case involved partial necrosis of a vastus lateralis myocutaneous flap due to excessive separation between the muscle and skin flap, which was corrected with additional full-thickness skin grafting. Deep infections of the flap were observed in 6 cases that required debridement. One limb experienced osteomyelitis recurrence and underwent bone reconstruction with a vascularised fibular bone graft. Donor site complications were noted in 1 patient with 2 flaps who was receiving anticoagulant therapy; this required haematoma evacuation and secondary suturing.

**Table 5:** Flap outcomes and postoperative complications.

Total number of limbs		13 limbs
Total number of flaps		14 flaps

Number of flaps that survived		14 flaps (100%)

Postoperative complication^a^	No complication	7 flaps (50%)
	Grade I	1 flap (7%)
	Grade II	0
	Grade IIIa	6 flaps (43%)
	Grade IIIb	0
	Grade IV	0

Systemic complication within 30 days		1 flap (asymptomatic DVT) (7%)

Flap-related complication	Complete flap loss	0
	Partial flap necrosis	2 flaps (14%)
	Arterial occlusion	0
	Venous occlusion	0
	Wound dehiscence	1 flap (7%)
	Postoperative infection	6 flaps (43%)
	Donor site problem	2 flaps (2 haematomas) (14%)

Additional surgery		9 flaps (64%)
	Debridement	6 flaps (43%)
	Other reconstruction	4 flaps (29%)
	- Bone graft	1 flap
	- Arthrodesis	1 flap
	- Skin graft	1 flap

Restoration of weight-bearing function		11 limbs (85%)
LEFS at peak postoperative condition^b^		46 (27–52)

Follow-up period (days)		692 (374–975)
Deceased at final follow-up		1 patient
Recurrence of minor foot ulcer		5 limbs (36%)

Data are presented as median (IQR).

**DVT**: Deep vein thrombosis; **LEFS**: Lower Extremity Functional Scale.

a According to the Clavien–Dindo classification.^27^

b The LEFS scores include cases that regained weight-bearing function, excluding one case with unilateral limb loss due to amputation on the opposite side.

Weight-bearing functionality was achieved in 11 patients with 11 limbs. The median follow-up period was 692 days. One patient died of acute myocardial infarction during the follow-up period; however, the patient had maintained a weight-bearing limb at the time of death. Kaplan–Meier analysis revealed an amputation-free survival rate of 100% at 1 year and 75% at 3 years. Among the surviving cases, those who achieved weight-bearing functionality continued to maintain it in their daily lives.

## Discussion

### Multidisciplinary approach and treatment algorithm

We introduced a treatment algorithm for refractory lower leg and foot infections in high-risk patients, involving a multidisciplinary team that included anaesthetists. This approach enabled us to perform limb salvage treatments while preventing complications associated with GA. In the 13 limbs of 11 patients reported here, we successfully avoided major amputation and achieved weight-bearing functionality in 85% of the cases, with no severe systemic complications. Previous studies have demonstrated a correlation between increased ASA grade and postoperative complications in free flap procedures.^10^^,^^11^ High-risk patients, including those with acute multiple trauma, poor general health, severe underlying conditions or obesity, are frequently excluded from long-duration GA.^2^^,^^26^ However, these patients often lack specific therapeutic options beyond careful systemic management.^6^^,^^13^ By incorporating a treatment algorithm that prioritises regional anaesthesia without GA for free flap procedures, we expanded the eligibility for reconstructive surgery in limb salvage treatments.

### Safety of CSE in preventing intraoperative hypotension

Renal failure, ASA class III or higher, prolonged surgeries, and hypoalbuminaemia have been identified as risk factors for severe perioperative systemic complications associated with GA.^9^ Additionally, elevated preoperative scores on indices such as the CCI, RCRI and ARISCAT PPS are associated with higher rates of postoperative cardiovascular and respiratory complications, often leading to recommendations to prevent GA.^22^^,^^23^ Using CSE for free flap surgeries without GA circumvents the complications that could adversely affect the systemic conditions and flap viability. These include hypoxia due to prolonged mechanical ventilation, blood pressure fluctuations, vascular spasms in the flap, ostoperative delirium and shivering. The placement of an epidural catheter further facilitates effective postoperative pain management.^8^^,^^15^^,^^16^

Intraoperative hypotension is a critical concern due to its association with fatal complications such as acute myocardial infarction and acute renal failure.^14^ Managing haemodynamics and pain during prolonged free flap surgeries under GA is particularly challenging in patients with poor systemic conditions.^12^^,^^27^ In our cohort, CSE without GA enabled stable haemodynamics with minimal need for vasopressors during long-duration free flap surgeries in high-risk patients.

Reports of free flap procedures performed solely under regional anaesthesia remain scarce.^15^^,^^16^^,^^28^ The prolonged nature of free flap surgeries, often necessitated by challenges in vascular anastomosis, and concerns about the efficacy of regional anaesthesia, are significant deterrents for anaesthetists and reconstructive surgeons.^16^ Although early reports indicate that epidural anaesthesia might reduce blood flow to denervated flaps due to a ‘steal phenomenon’ caused by decreased vascular resistance in sympathetically blocked peripheral tissues,^29^^,^^30^ several recent studies indicate that epidural anaesthesia may benefit free flap procedures.^12^^,^^31^ By employing CSE, which provides more reliable anaesthesia than epidural anaesthesia alone,^15^ we successfully conducted surgeries lasting up to 9.5 h. This achievement underscores the importance of mutual understanding between anaesthetists and reconstructive surgeons in using regional anaesthesia alone for free flap procedures.

### Treatment planning with anaesthetists

Effective treatment planning with anaesthetists is essential in selecting the optimal flap type when considering anaesthesia methods and potential donor site complications. For high-risk cases with infected lower limbs, poor local blood flow often necessitates the use of fasciocutaneous flaps to minimise the risk of a steal phenomenon.^32^^,^^33^ Although the ALT flap was most commonly used due to its feasibility under CSE, it is critical to confirm that the ALT's nutrient vessels are not involved in the collateral circulation of the affected limb.^34^

In cases of recalcitrant calcaneal osteomyelitis, our standard treatment involves thorough curettage of the calcaneal medullary cavity and filling it with a muscle flap, consistent with the method described by Ghod et al.^35^ In 1 case, the anaesthetist recommended altering the treatment plan due to the high risk associated with GA for latissimus dorsi flap elevation. Instead, the vastus lateralis myocutaneous flap was elevated under CSE, enabling successful limb salvage without systemic complications. The selection of the donor site is typically based on the ease of flap elevation and vascular anastomosis. However, preparation to switch sides based on the efficacy of the epidural anaesthesia is essential for ensuring procedural success.

### Vascular anastomosis for severely atherosclerotic and calcified vessels

Vascular calcification presents a significant challenge in free flap surgery for high-risk lower limb cases.^3^^,^^7^^,^^36^ Some reports suggest using less calcified, smaller perforator branches as recipient vessels.^5^^,^^34^ However, the survival area of the flap is dependent on the blood flow from the recipient vessel, making the use of the main artery preferable for larger flap survival.^37^ In limb salvage treatment, it is crucial to preserve blood flow to the peripheral tissues, making the ETS technique essential. Furthermore, a large slit is most useful for anastomosis to calcified recipient vessels.^3^^,^^7^ We use the parachute technique, which facilitates easy and secure closure of leak-prone defects at the heel.^19^^,^^20^ Calcification is often observed in the nutrient vessels of ALT flaps as well.^1^ Although Black et al.^7^ reported the usefulness of interposing a vein when the flap and recipient vessels are calcified, we select the area where calcification of the dorsalis pedis artery is absent on the dorsal aspect of the ankle as the preferred recipient vessel to avoid complex vein grafting (Figure 4).

### Functional outcomes and amputation-free survival

For the long-term preservation of residual limb function, postoperative care, including foot care and orthotic management, is essential. Five limbs developed recurrent ulcers, but major amputation was avoided during the survival period. In cases where weight-bearing function was achieved, our outcomes in terms of LEFS scores (46 out of 80) were consistent with those reported by Lu et al. (46 out of 80).^2^ Despite its low-demand nature, weight-bearing function was maintained throughout the survival period. The amputation-free survival rate in this study was slightly lower than those reported by Chou et al.^1^ (92.3% at 1 year and 88.4% at 5 years), Oh et al.^4^ (84.9% at 53 months), and Huang et al.^36^ (96% at 1 year and 92% at 2 years), which may reflect the inclusion of a cohort with more severe complications compared to those in previous studies.

### Considerations when implementing CSE

When implementing CSE, attention to the following points is crucial to prevent complications:

1. Patients with active cardiac conditions such as unstable coronary artery disease, hypertrophic cardiomyopathy, severe arrhythmias or advanced valvular disease may be at risk of circulatory collapse with regional anaesthesia administration. Therefore, a thorough evaluation by a cardiologist regarding procedural tolerance is essential.^38^
2. During lumbar puncture for CSE, it is necessary to prevent complications such as epidural haematoma.^39^ Although the incidence is relatively low (approximately 1 in 4000 cases), it increases with anticoagulant use, which are often necessary in high-risk patients, including those on chronic antiplatelet therapy or undergoing dialysis. Therefore, careful management of anticoagulation and vigilant neurological monitoring post-procedure are essential.
3. CSE involves prolonged awake surgery, necessitating patient cooperation throughout. As noted in previous reports describing cases where patients enjoyed watching films or playing games on tablets during surgery,^16^ anaesthetists and reconstructive surgeons ensure that patients are informed about the procedure and reassured of a relaxed, pain-free experience throughout the operation. It is recommended to discuss anaesthesia methods and expectations with the patient preoperatively and to consider adding sedation if the patient wishes.

### Limitation

This study has some limitations: it is a retrospective analysis with a small sample size and short follow-up period. Additionally, needle-through-needle CSE is technically challenging and should only be performed by experienced anaesthetists.^40^ Future studies with larger sample sizes are needed to validate the algorithm's effectiveness and address potential issues.

## Conclusion

In this study, we reported the treatment outcomes of limb salvage procedures in patients with high-risk, refractory lower leg and foot infections based on a multidisciplinary team approach, including anaesthetists, at our institution. For the 12 patients with 13 limbs where infection control was achieved, free flap reconstruction was performed using regional anaesthesia alone, thereby preventing GA and severe systemic complications, and preserving the limbs in all cases. Promoting the use of free flap procedures under regional anaesthesia without GA to reconstructive surgeons and anaesthetists can help expand the indications for limb salvage treatments and prevent major amputations.

## Conflicts of interest

None.

## References

[1] Chou C., Kuo P.J., Chen Y.C. (2016). Combination of vascular intervention surgery and free tissue transfer for critical diabetic limb salvage. Ann Plast Surg.

[2] Lu J., DeFazio M.V., Lakhiani C. (2019). Limb salvage and functional outcomes following free tissue transfer for the treatment of recalcitrant diabetic foot ulcers. J Reconstr Microsurg.

[3] Nigam M., Zolper E.G., Sharif-Askary B. (2022). Expanding criteria for limb salvage in comorbid patients with nonhealing wounds: the MedStar Georgetown protocol and lessons learned after 200 lower extremity free flaps. Plast Reconstr Surg.

[4] Oh T.S., Lee H.S., Hong J.P. (2013). Diabetic foot reconstruction using free flaps increases 5-year-survival rate. J Plast Reconstr Aesthet Surg.

[5] Salibian A.A., Swerdlow M.A., Kondra K., Patel K.M. (2024). Extreme limb salvage: the thin SCIP flap for distal amputation coverage in highly comorbid patients. Plast Reconstr Surg.

[6] Serletti J.M., Higgins J.P., Moran S., Orlando G.S. (2000). Factors affecting outcome in free-tissue transfer in the elderly. Plast Reconstr Surg.

[7] Black C., Fan K.L., Defazio M.V. (2020). Limb salvage rates and functional outcomes using a longitudinal slit arteriotomy end-to-side anastomosis for limb-threatening defects in a high-risk patient population. Plast Reconstr Surg.

[8] Memtsoudis S.G., Cozowicz C., Bekeris J. (2019). Anaesthetic care of patients undergoing primary hip and knee arthroplasty: consensus recommendations from the International Consensus on Anaesthesia-related outcomes after surgery group (ICAROS) based on a systematic review and meta-analysis. Br J Anaesth.

[9] Rujirojindakul P., Geater A.F., McNeil E.B. (2012). Risk factors for reintubation in the post-anaesthetic care unit: a case-control study. Br J Anaesth.

[10] Musharrafieh R.S., Saghieh S., Macari G., Atiyeh B. (2003). Diabetic foot salvage with microsurgical free-tissue transfer. Microsurgery.

[11] Oishi S.N., Levin L.S., Pederson W.C. (1993). Microsurgical management of extremity wounds in diabetics with peripheral vascular disease. Plast Reconstr Surg.

[12] Hagau N., Longrois D. (2009). Anesthesia for free vascularized tissue transfer. Microsurgery.

[13] Lese I., Biedermann R., Constantinescu M., Grobbelaar A.O., Olariu R. (2021). Predicting risk factors that lead to free flap failure and vascular compromise: a single unit experience with 565 free tissue transfers. J Plast Reconstr Aesthet Surg.

[14] Liu Y., Su M., Li W., Yuan H., Yang C. (2019). Comparison of general anesthesia with endotracheal intubation, combined spinal-epidural anesthesia, and general anesthesia with laryngeal mask airway and nerve block for intertrochanteric fracture surgeries in elderly patients: a retrospective cohort study. BMC Anesthesiol.

[15] Ciudad P., Escandon J.M., Manrique O.J., Escobar H., Pejerrey Mago B., Arredondo Malca A. (2023). Efficacy of combined spinal-epidural anesthesia for lower extremity microvascular reconstruction. J Surg Res.

[16] Galitzine S., Wilson K., Edington M., Burumdayal A., McNally M. (2021). Patients' reported experiences and outcomes following surgical excision of lower limb osteomyelitis and microvascular free tissue reconstruction under 'awake' epidural anaesthesia and sedation. Surgeon.

[17] Doyle D.J., Goyal A., Bansal P., Garmon E.H. (2021). StatPearls.

[18] Stocks G.M., Hallworth S.P., Fernando R. (2000). Evaluation of a spinal needle locking device for use with the combined spinal epidural technique. Anaesthesia.

[19] Motomiya M., Watanabe N., Kawamura D., Yasui K., Adachi A., Iwasaki N. (2020). Reliable free flaps using the microscopic parachute end-to-side technique in severe extremity injuries. J Plast Reconstr Aesthet Surg.

[20] Motomiya M., Watanabe N., Nakamura S., Kameda Y., Kawamura D., Iwasaki N. (2021). Blood flow distribution after end-to-side anastomosis with wide arteriotomy in extremity free flap surgery. J Plast Reconstr Aesthet Surg.

[21] Charlson M.E., Pompei P., Ales K.L., MacKenzie C.R. (1987). A new method of classifying prognostic comorbidity in longitudinal studies: development and validation. J Infect Dis.

[22] Hiraoka E., Tanabe K., Izuta S. (2023). JCS 2022 Guideline on perioperative cardiovascular assessment and management for non-cardiac surgery. Circ J.

[23] Canet J., Gallart L., Gomar C. (2010). Prediction of postoperative pulmonary complications in a population-based surgical cohort. Anesthesiology.

[24] Ferraresi R., Ucci A., Pizzuto A. (2021). A novel scoring system for small artery disease and medial arterial calcification is strongly associated with major adverse limb events in patients with chronic limb-threatening ischemia. J Endovasc Ther.

[25] Clavien P.A., Barkun J., de Oliveira M.L. (2009). The Clavien-Dindo classification of surgical complications: five-year experience. Ann Surg.

[26] Li R., Liu L., Wei K., Zheng X., Zeng J., Chen Q. (2023). Effect of noninvasive respiratory support after extubation on postoperative pulmonary complications in obese patients: a systematic review and network meta-analysis. J Clin Anesth.

[27] Motakef S., Mountziaris P.M., Ismail I.K., Agag R.L., Patel A. (2015). Emerging paradigms in perioperative management for microsurgical free tissue transfer: review of the literature and evidence-based guidelines. Plast Reconstr Surg.

[28] Alam N.H., Haeney J.A., Platt A.J. (2006). Three episodes of gracilis free muscle transfer under epidural anaesthesia. J Plast Reconstr Aesthet Surg.

[29] Erni D., Banic A., Signer C., Sigurdsson G.H. (1999). Effects of epidural anaesthesia on microcirculatory blood flow in free flaps in patients under general anaesthesia. Eur J Anaesthesiol.

[30] van Twisk R., Gielen M.J., Pavlov P.W., Robinson P.H. (1988). Is additional epidural sympathetic block in microvascular surgery contraindicated? A preliminary report. Br J Plast Surg.

[31] Park J.W., Moon J., Lee K.T. (2020). Comparison of surgical outcomes of free flap reconstructions performed by expert microsurgeons and trainees who completed a structured microsurgical training program in a teaching hospital. J Plast Reconstr Aesthet Surg.

[32] Rainer C., Schwabegger A.H., Meirer R., Perkmann R., Ninkovic M., Ninkovic M. (2003). Microsurgical management of the diabetic foot. J Reconstr Microsurg.

[33] Sonntag B.V., Murphy R.X., Chernofsky M.A., Chowdary R.P (1995). Microvascular steal phenomenon in lower extremity reconstruction. Ann Plast Surg.

[34] Suh H.P., Kim Y., Suh Y., Hong J. (2018). Multidetector computed tomography (CT) analysis of 168 cases in diabetic patients with total superficial femoral artery occlusion: is it safe to use an anterolateral thigh flap without CT angiography in diabetic patients?. J Reconstr Microsurg.

[35] Ghods M., Grabs R., Kersten C., Chatzopoulos P.P., Geomelas M. (2012). A modified free muscle transfer technique to effectively treat chronic and persistent calcaneal osteomyelitis. Ann Plast Surg.

[36] Huang C.C., Chang C.H., Hsu H. (2014). Endovascular revascularization and free tissue transfer for lower limb salvage. J Plast Reconstr Aesthet Surg.

[37] Miyamoto S., Minabe T., Harii K. (2008). Effect of recipient arterial blood inflow on free flap survival area. Plast Reconstr Surg.

[38] Vigoda M.M., Sweitzer B., Miljkovic N. (2011). 2007 American College of Cardiology/American Heart Association (ACC/AHA) Guidelines on perioperative cardiac evaluation are usually incorrectly applied by anesthesiology residents evaluating simulated patients. Anesth Analg.

[39] Stafford-Smith M. (1996). Impaired haemostasis and regional anaesthesia. Can J Anaesth.

[40] Rawal N., Holmström B., Crowhurst J.A., Van Zundert A. (2000). The combined spinal-epidural technique. Anesthesiol Clin North Am.

